# Effect of Intervention in Subjects with High Risk of Diabetes Mellitus in Pakistan

**DOI:** 10.1155/2012/867604

**Published:** 2012-07-19

**Authors:** Muhammad Zafar Iqbal Hydrie, Abdul Basit, A. Samad Shera, Akhtar Hussain

**Affiliations:** ^1^Section of International Community Health, Department of Community Medicine, Institute of Health and Society, Faculty of Medicine, University of Oslo, P.O. Box 1130, Blindern, Oslo, Norway; ^2^Department of Medicine, Baqai Institute of Diabetology and Endocrinology, Baqai Medical University, Plot No. 1-2, II-B, Block 2, Nazimabad, Karachi 74600, Pakistan; ^3^WHO Collaborating Centre, Diabetic Association of Pakistan, 5-E/3, Nazimabad, Karachi 74600, Pakistan

## Abstract

*Aims*. To observe the rate of conversion from impaired glucose tolerance (IGT) to diabetes following lifestyle modification (LSM) or a combination of lifestyle and metformin compared to a control population with 18-month followup. *Methods*. Forty screening camps were organized, which 5000 people attended. Around 2300 persons filled the questionnaire and 1825 subjects were identified as high risk. Of 1739 subjects who took the oral glucose tolerance test, 317 subjects were identified as IGT. The 317 IGT subjects were randomized into three groups: control group was given standard medical advice, LSM group was given intensive lifestyle modification advice, while LSM + drug group was given intensive lifestyle advice and metformin 500 mg twice daily. *Results*. At the end, 273 subjects completed the study, giving a compliance rate of 86%. Total of 47 incident cases of diabetes were diagnosed (overall incidence was 4 cases per 1000 person-months with the incidence of 8.6 cases in control group, 2.5 cases in the LSM, and 2.3 cases in the LSM + drug groups). *Conclusions*. Study showed that lifestyle intervention had a major impact in preventing diabetes among IGT subjects in this region. Adding drug did not show any improved results. We recommend lifestyle advice and followup should be incorporated in primary health care.

## 1. Introduction

The prevalence of diabetes is increasing globally and this burden of disease is one of the most challenging public health problems of the 21st century with Asia as the epicentre [1, 2]. It is increasing in epidemic proportions with one person developing diabetes every five seconds globally [1, 2]. Once diabetes develops, it causes disability, increased health costs to the person, and reduced life expectancy with someone dying from diabetes every ten seconds in the world [2]. Thus, diabetes is a chronic debilitating disease causing life-long complications such as heart disease, blindness, kidney damage, and foot amputations. The most dramatic increase in type 2 diabetes is occurring in genetically predisposed populations due to major lifestyle transitions that are taking place. These include changes in diet and reduction in physical activity, with consequent increase in the prevalence of obesity leading to an increased burden of diabetes [2, 3].

Randomized controlled trials have shown that progression to diabetes can be reduced in people at identifiable risk through interventions [2–4]. Thus evidence from clinical trials suggest that subjects at risk of developing diabetes can prevent or delay the onset of type 2 diabetes by lifestyle modification or medication [2, 3]. A number of studies done in China, Finland, USA, and India have demonstrated the importance of healthier lifestyle in preventing or reducing the occurrence of diabetes [3, 5–9]. The collective results of such prevention studies showed an average reduction of 51% in new cases of diabetes [4]. Studies from Finland and USA showed that the most powerful way to prevent the occurrence of diabetes was to modify lifestyle conducive to improved metabolic health [6–8].

Most intervention studies targeted diabetes prevention by achieving and maintaining a healthy body weight through a combination of dietary measures and physical activity in high-risk subjects [2]. However, based on genetics and defining cardiometabolic state including level of obesity, fat deposition pattern, and dietary habits in different populations, this intervention strategy may need to be revised. For example, different ethnic groups have different body mass index (BMI) cutoffs which may have a varied effect on intervention as evident in the Indian study where risk reduction rate of 28% was seen compared to 58% risk reduction of diabetes in the Finnish and USA studies [7–10].

One limitation of the study in India was that the subjects were recruited from the local railway company and most of them were vegetarians. South Asians are a heterogenous group based on different religious and cultural practices including food habits and they are genetically different. Studies have also noted dietary differences within the ethnic groups in South Asians [11, 12]. For example, the diet of South Asians (mainly Punjabi) studied in Scotland was found to have large differences between Muslim and non-Muslim groups, with Muslims more likely to eat meat and less likely to eat fruit and cooked vegetables than non-Muslims [13]. This difference was also seen in another study between Muslims and Hindus (probably due to their vegetarianism) [14]. This difference in diet due to ethnicity and religion may have an effect on intervention strategies and needs to be further explored to better understand the effect of intervention programs in these communities.

Therefore, we conducted this intervention study in the largest city (Karachi) of Pakistan amongst the general population. The main aim of the study was to observe the rate of conversion from IGT to diabetes following lifestyle modification program and a combination of lifestyle modification and oral hypoglycaemic agent (metformin) compared to a control population with 18-month followup.

## 2. Methodology

### 2.1. Location

Karachi is the largest and most populous city of Pakistan. All the major ethnic groups of the country reside here with Muhajirs forming the dominant ethnic group in Karachi. Our primary prevention team visited different primary health care centres within the city to generate awareness and distributed educational leaflets about our primary prevention program.

### 2.2. Study Procedure

A number of strategies starting with opportunistic screening at 2 different outpatient clinics were adopted to secure participation of the general population and create awareness about diabetes prevention. With the aim to reach a larger audience, our diabetes prevention team arranged a series of 2-day awareness lectures at various places in the city by going to offices, service organizations, factories, and visiting health care centres. Lectures on diabetes and its prevention were delivered by the prevention team in local language to the audience on the first day. The audience were asked to fill a risk questionnaire at the end of the first day. On the second day, screening of high-risk subjects was done according to the results of the questionnaire and all high-risk subjects were invited for an OGTT. During the time OGTT was done, all the subjects underwent a detailed anthropometric and medical examination and were been asked about their sociodemographic, physical activities, and dietary habits including information on quantity and quality of meals by a dietician and physical trainer.

### 2.3. Design of the Study

This was a randomized controlled clinical trial (RCT) conducted in subjects over 30 years of age who were diagnosed as having IGT according to World Health Organization criteria [15]. The IGT subjects were followed for a period of 18 months prospectively.

A questionnaire was filled as the first step to identify subjects at an increased risk of developing type 2 diabetes from the overall population. This standardized questionnaire included (a) family history (parents or siblings with diabetes), (b) high body mass index, (c) low-physical activity, (d) age, (e) hypertension, (f) high cholesterol or triglycerides, (g) history of gestational diabetes or birth weight >3.5 kgs. Those identified as high risk (*n* = 1825) were requested to undergo an oral glucose tolerance test (OGTT). Of these 1739 came for the OGTT giving a response rate of 95.3%. Subjects with 2-hour glucose levels between 140 to 199 mg/dL were identified as having impaired glucose tolerance (IGT) and were requested to participate in the study. Those who were diagnosed with diabetes (*n* = 181) were referred to respective hospitals for further medical care.

**Figure 1:**
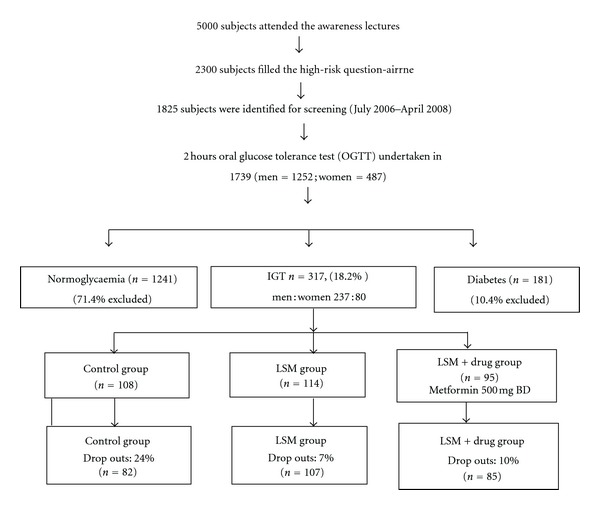
Flowchart with recruitment of persons for the oral glucose tolerance test (OGTT), screening, and randomization.

After taking informed consent, the participants were randomized by age strata (31–40 years, 41–50 years, 51–60 years, and >60 years) into three different arms. This was to ensure equal age representation in all the arms of the intervention. The three groups as shown in the flowchart were followed for 18 months (Figure 1). First group was given standard medical advice (control group), second group was given intensive lifestyle modification advice (LSM group) while third group was given intensive lifestyle modification advice and metformin (LSM + drug group).

### 2.4. Subjects

An estimated 5000 people attended the diabetes prevention lectures and visited the screening camps. Of these 2300 filled a high-risk questionairne and 1825 were identified as high-risk subjects which were requested to undertake a standardized oral glucose tolerance test (OGTT). Of these 1739 agreed to undertake the OGTT and 317 subjects were identified as having impaired glucose tolerance (IGT group).

Of those who underwent OGTT, 72% were males, 10.4% were found to have diabetes, and 18% were having impaired glucose tolerance (IGT). Baseline characteristics of the subjects who underwent OGTT showed an increasing trend in terms of age, BMI, and blood pressure from normal glucose tolerance (NGT) to diabetes (DM) subjects as shown in Table 1.

### 2.5. Investigations

All IGT subjects had fasting lipid profile, fasting insulin levels, OGTT, and HbA1c done at 0, 9, and 18 months. At the interim 9-month visit, confirmation of diabetes was made with OGTT. Plasma glucose was measured using the glucose oxidase-peroxidase method. The fasting serum lipid profile was estimated using standard enzymatic procedures. HbA1c was measured by HPLC using Bio-Rad, a procedure certified by the National Glycohemoglobin Standardization Program. Weight, height, waist circumference, and blood pressure were measured at each scheduled visit. All the subjects in the IGT cohort were seen after the OGTT tests and randomized into one of the three groups at baseline (0 week). They were followed according to their assigned groups and seen every two months by the primary prevention team. At 9 and 18 months blood tests were done to confirm their glucose status and assess their biochemical parameters. Weight and height were measured with the subjects minimally clothed, without shoes, and in a standing position. Waist circumference was measured at the midpoint between the iliac crest and the costal arch. Blood pressure was measured twice, 5 min apart, in a sitting position, and the average of the two readings was recorded.

### 2.6. Intervention and Randomization

All the subjects who were identified as IGT and agreed to participate in the study were randomized into three groups as shown in Figure 1. The subjects assigned to their respective groups were followed till the end of the study.

Training sessions and counseling were given to subjects in the intervention group about achieving the intervention goals, which included reduction of ≥5% of body weight loss via diet control and physical exercise, total fat intake less than 30% of energy consumed, fiber intake of 15 g/1000 kcal, and moderate exercise for minimum 30 min/day. Frequent ingestion of wholemeal products, vegetables and fruits, low-fat milk, meat products, and vegetable oils rich in monounsaturated fatty acids was recommended. The subjects had sessions with a dietician and a physical trainer at each visit and they were individually counselled to increase their level of physical activity. Endurance exercises such as walking, jogging, and cycling were recommended to improve fitness. Reinforcing behaviour modification was done by giving advice on healthy diet and physical activity to each subject in consequent sessions. These interventions were based on supervised, individually tailored training advice which was offered to improve the physical fitness of each individual.

Subjects in the control group were given general diet and exercise information at baseline and followed at subsequent visits, but no intensive individual specific counselling was given to them. While subjects in the intervention + drug groups were also seen every 2 months by a medical doctor for their drug adherence.

### 2.7. Followup

Reinforcement and counselling were done in all groups every 2 months, with the intensive group seen by the medical officer, dietician, and physical trainer, while the control group was seen by the medical officer as described in detail elsewhere [16].

### 2.8. Primary Outcome

The primary outcome was defined as developing diabetes indicated by either fasting plasma glucose of >125 mg/dL and/or 2-hour plasma glucose of >199 mg/dL confirmed at 9- and 18-months followup by an OGTT [15]. Subjects identified as having diabetes were excluded from the study and given medical advice with referral to physicians for further followup.

## 3. Statistics

Mean and standard deviation were reported for continuous variables and intergroup comparisons were tested by two-tailed ANOVA. Comparison of proportions was by *χ*2 analysis. The proportion of subjects developing diabetes in each group and their comparison was by *χ*2 analysis.

For the intervention measures, the absolute and relative risk reductions, 95% CIs of the estimates, and the number needed to treat to prevent diabetes in one person were calculated. A *P* value <0.05 was considered significant. The statistical package SPSS (PASW Statistics 18) was used for analyses.

## 4. Results

The 317 IGT subjects were randomized into three groups as shown in Table 2. More than half (56%) of the subjects were between 30 and 44 years of age in the IGT cohort and 36% of the subjects were unskilled/skilled manual labourers. Positive family history of diabetes, hypertension, cardiovascular disease, and stroke was present in 49%, 38%, 31%, and 17% of the subjects respectively while 25% had hypertension at the start of the study.

**Figure 2:**
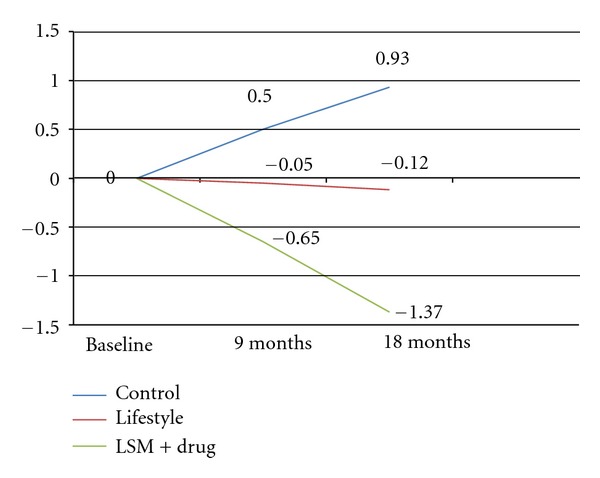
Weight changes in 18 months. (Significant difference between the Control and LSM + drug groups: *P* value = 0.003).

**Figure 3:**
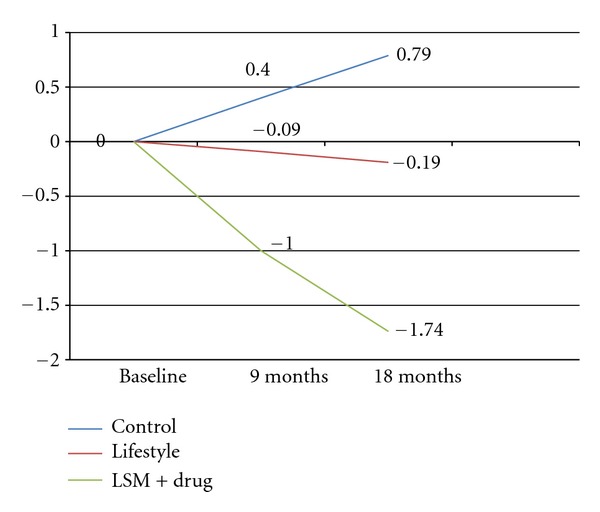
Waist circumference. (No significant difference seen between the three groups).

During the course of the study, the mean body weight and waist circumference decreased in the lifestyle and LSM + drug groups while it increased in the control group as shown in Figures 2 and 3.

At the end of the study, 273 subjects completed the study giving a response rate of 86%. A total of 47 incident cases of diabetes were diagnosed during the study: 19 cases at 9 months and 28 cases at 18 months or closure of the study. The overall incidence of diabetes was 4 cases with 8.6 cases in the control group, 2.5 cases in the life style modification (LSM) group, and 2.3 cases per 1000 person-months in the LSM + drug group as shown in Table 3. The numbers to be treated to prevent one incident case of diabetes was 9 and 8 in lifestyle and LSM + drug groups, respectively.

### 4.1. Adverse Events

Overall 44 subjects dropped out or were lost to followup. In the control group there were 2 deaths while 24 subjects dropped out during the study. In the lifestyle modification group 8 subjects refused to continue the study and dropped out. In the LSM + drug group 5 subjects stopped taking the drug either due to side effects of the drug such as gastrointestinal problems or complaining of weakness probably due to hypoglycemia while 5 subjects refused to follow due to personal reasons and were lost to followup.

## 5. Discussion

Our data suggest that lifestyle intervention is highly effective in preventing high-risk individuals (IGT) from conversion to diabetes in this population. Adding oral hypoglycaemic agent (metformin) in addition to lifestyle modification was not found to be advantageous for the prevention. Our data is in line with the previous studies done in other population [6–9].

The progression rate of IGT to diabetes in our control subjects was lower compared to Indian and Chinese controls (8.2% per 12 months compared to 18.3% and 11.3% per 12 months, resp.) [5, 9]. But this was significantly higher than seen in the Finnish (6% per year) and DPP (11 per 100 person-years) studies [5, 7, 8]. All subjects in the control group also received general health advice about diet, nutrition, and exercise at baseline and at subsequent follow-up visits. This may have helped to increase their awareness about their diabetes risk, and some subjects may have benefited from the advice or made subsequent lifestyle modifications on this basis. Absolute-risk reduction of 10.7/100 was seen in the intensive lifestyle group, which was greater than seen in IDPP (15.7/100) [9]. More number of subjects were needed to treat in order to prevent one case of diabetes in the intensive group (9 versus 6.4) in our study compared to IDPP [9].

Baseline characteristics of our subjects show similar mean age as other Asian studies (mean age Indian 45.9 ± 5.7 years, Chinese 45 ± 9.1 years and ours 43.6 ± 9.9 years) but had comparatively higher BMI (kg/m^2^) (Indian 25.8 ± 3.5, Chinese 25.8 ± 3.8 and ours 27.1 ± 5.0) [5, 9]. However, our subjects were still younger and leaner compared to the Finnish (age 55 ± 7.0 years, BMI 31 ± 4.6) and the American subjects (age 50.6 ± 10.7 years, BMI 34 ± 6.7) [6, 8]. The follow-up period in our study was nearly half in duration (only 18 months) compared to the Indian, American, and Finnish studies [6–8].

In our study, the progression of IGT to diabetes was comparable to other Asian studies, and it showed the effectiveness of lifestyle modification involving moderate physical activity and diet modification to prevent diabetes in this population. Thus, making lifestyle modifications could have beneficial impact on the whole community. Adding metformin had an additional benefit but its impact was quite small with the relative-risk reduction of 5.5%, from 71% to 76.5% in the lifestyle modification + drug group in our study. This difference was not found to be statistically significant.

Our results showed that to reduce the burden of diabetes epidemic, effective primary prevention can be achieved through lifestyle modification. Therefore, it is suggested that necessary policy development on the prevention of diabetes should emphasize on the lifestyle modification. Recent updates from the China DaQing Prevention Study, the Finnish Diabetes Prevention Study, the American Diabetes Prevention Program Outcome Study, and the Look Ahead Study have all shown that the most efficient way to manage diabetes and its complications is to prevent diabetes in the first place. This in turn has led to policy documents from expert organizations such as the Disease Control Priorities Project (DCP-2), the European Society of Cardiology and European Association for the Study of Diabetes, the Canadian Diabetes Association, the American Diabetes Association, and the International Diabetes Federation, all recommending lifestyle changes such as weight loss and increased physical activity for the prevention of T2DM among those with prediabetes [2, 9, 17–21].

The main motivation for the prevention of type 2 diabetes is that it can prevent or delay the onset of diabetes and its complications, thereby reducing the life entrenched financial burden and unnecessary human sufferings of diabetes on both the individual and on the society at large. Developing countries have to face this additional burden on their already ailing economy [22, 23]; therefore, primary prevention programs need to become an integral part of Primary Health Services and strategies for reducing the diabetes burden at all levels.

## Figures and Tables

**Table 1:** Baseline characteristics of 1739 identified high-risk subjects by questionnaire.

	NGT	IGT	DM	*P* value
*N*	1241	317	181	
Age of patient (years)	40.1 ± 8.9	43.6 ± 9.9	44.4 ± 9.7	<0.0001
Body mass index (kg/m^2^)	25.8 ± 5.3	27.1 ± 5.0	27.3 ± 5.2	<0.0001
Systolic BP (mmHg)	118.3 ± 16.5	121.5 ± 16.8	124.3 ± 17.7	<0.0001
Diastolic BP (mmHg)	82.5 ± 11.0	84.6 ± 10.9	85.4 ± 13.1	0.001

**Table 2:** Baseline characteristics of the 317 Subjects in the IGT Cohort.

	Control	Lifestyle	LSM + drug
*N*	108	114	95
Age in years	44.2 ± 10.9	43.1 ± 10.1	43.5 ± 8.4
Body mass index (kg/m^2^)	27.0 ± 5.7	26.1 ± 4.7	28.1 ± 4.3
Systolic BP (mmHg)	121 ± 17	123 ± 19	120 ± 14
Diastolic BP(mmHg)	84 ± 11	86 ± 12	84 ± 9
Cholesterol (mg/dL)	179.1 ± 37	178.6 ± 34	180.0 ± 36
Triglycerides (mg/dL)	153.4 ± 109	147.3 ± 86	171.5 ± 119
HDL-C (mg/dL)	37.8 ± 4.3	37.4 ± 4.5	37.8 ± 7.8
LDL-C (mg/dL)	117.2 ± 25.1	116.5 ± 22.7	117.0 ± 24.6

**Table 3:** Comparison of the outcome at 18 months in the three groups.

	Control	Lifestyle	LSM + drug
*N*	82	107	85
Cases per 1000 person-months	8.6	2.5	2.3
Absolute-risk reduction %		10.7	11.5
Relative-risk reduction % (95% CI)		71 (13.7–90.3)	76.5 (19.7–93.1)
NNT for 18 months to prevent DM in one case		9	8
